# Computed Tomography of the Chest in Younger Pediatric Patients with Thoracic Blunt Trauma Rarely Changes Surgical Management

**DOI:** 10.5811/westjem.2022.2.54094

**Published:** 2022-05-02

**Authors:** Irma T. Ugalde, Hei Kit Chan, Donna Mendez, Henry E. Wang

**Affiliations:** * McGovern Medical School at UTHealth Houston, Department of Emergency Medicine, Houston, Texas; † The Ohio State University, Department of Emergency Medicine, Columbus, Ohio

## Abstract

**Introduction:**

Thoracic trauma is the second leading cause of death after traumatic brain injury in children presenting with blunt chest trauma, which represents 80% of thoracic trauma in children. We hypothesized that older children undergo more clinical and surgical changes in management than younger children screened for intrathoracic injury at a single, urban, pediatric Level I trauma center.

**Methods:**

In this retrospective observational study, we determined the frequencies and types of lesions diagnosed only by chest computed tomography (CCT) and resulting changes of clinical and surgical management among different age groups in a pediatric cohort examined for blunt trauma with chest radiograph and CCT. We used logistic regression to quantify variations in CCT diagnoses and changes in clinical and surgical management across age groups. For each age category, we determined the odds ratio for diagnosis made only on CCT and subsequent changes in all clinical management and, specifically, surgical management. We performed the test of trend to determine the relationship across age with changes in management resulting from additional diagnoses made by CCT.

**Results:**

We analyzed data on 1,235 patients screened for intrathoracic injury. We found the following overall clinical management and surgical management changes, respectively, per age group: 0–2 years, 5/128 (3.9) and 0/128 (0.0); 3–6 years, 11/212 (5.2) and 1/212 (0.5); 7–10 years, 16/175 (9.1) and 2/175 (1.1); 11–13 years, 17/188 (9.0) and 3/188 (1.6); 14–17 years, 58/532 (10.9) and 25/532 (4.7). There were no observed surgical management changes in the 0–2 age group and, thus, no estimated odds ratio could be calculated. The adjusted odds ratios for the occurrence of surgical change in management (14–17 age group as reference) was 0.1 (0.0–0.9) for 3–6 years, 0.3 (0.1–1.3) for 7–10 years, and 0.3 (0.1–1.1) for 11–13 years. The trend of odds ratios across ages showed that with every subsequent year of life there was a 10% increase in management change and a 30% increase in surgical management change.

**Conclusion:**

Chest computed tomography plays a limited role in younger children and seldom significantly changes management albeit making additional diagnoses.

## INTRODUCTION

Trauma is the leading cause of morbidity and mortality in children. While thoracic trauma occurs in about 5–12% of children admitted to the hospital with trauma, it has a high morbidity and mortality.^1^ Advanced Trauma Life Support guidelines recommend chest radiograph (CXR) as the initial diagnostic imaging modality for the evaluation of thoracic trauma.^2^,^3^ Computed tomography of the chest (CCT) is still widely used in the evaluation of trauma patients. The hypothetical benefits of speed, convenience, and anatomic detail provided by CCT have made it an appealing diagnostic and screening choice for several decades.^4^ The actual radiation exposure, cost, and its contribution to an increased length of stay (LOS) in the emergency department (ED) are real considerations that must be weighed and limit the use of CCT in children when feasible.^5^,^6^

A decision instrument has been derived and validated for when to obtain a CCT in trauma patients ≥15 years.^7^,^8^ In younger children, clinical prediction rules and guidelines assist clinicians with regard to suitable utilization of CCT in blunt trauma, but these are not validated.^9^–^14^ Studies demonstrate implementation of these guidelines can decrease CT utilization for trauma; however, adoption at individual centers, even pediatric trauma centers, varies.^15^,^16^ Children are more likely to undergo a CT if they are older, more severely injured (higher Injury Severity Score [ISS]), have a lower Glasgow Coma Scale (GCS), motor vehicle collision (MVC) injury as a mechanism, or have severe head, chest, or abdominal injuries.^17^ What is known to a lesser degree is how often current imaging of children leads to abnormal findings on CCT that lead to significant procedural interventions or poor outcome and how that outcome is associated with pediatric subgroups by age.

### Goals of the Study

We hypothesized that older children would undergo more clinical and surgical changes in management than younger children screened for intrathoracic injury at a single, urban, pediatric Level I trauma center when CCT identified an injury not demonstrated on CXR.

## METHODS

### Study Design and Setting

We analyzed data from a retrospective observational study of pediatric trauma. Our local institutional review board (IRB) approved the study. We performed a retrospective analysis of children treated at Children’s Memorial Hermann Hospital in Houston, TX, from 2009–2015. Memorial Hermann Hospital Medical Center is the only American College of Surgeons-verified Level I adult *and* pediatric trauma center in Houston. Annually, the center sees about 6000 trauma patients ≥16 years and about 1200 trauma patients 15 years and younger.

### Patients

We included patients <18 years evaluated with CXR followed by CCT within 24 hours of blunt trauma. Therefore, all patients had a gold standard comparison to the CXR and represented all patients screened for thoracic trauma at our institution. Patient data elements were queried from the institutional trauma registry. The trauma registry uses as its inclusion criteria the National Trauma Data Standard Data Dictionary, which includes the presence of *International Classification of Diseases*, revisions 9 and 10 (ICD9/ICD10) codable injury presenting within 14 days of the injury (and excludes patients whose injuries were only isolated and superficial such as abrasions and soft tissue contusions). In addition, patients must meet one of the following criteria: admitted patients (observation or inpatient); dead on arrival/died in the ED; transfer out of the ED for higher level of care; or transfer in from an acute care ED/hospital regardless of whether they were discharged from the ED or admitted to the hospital.

**Population Health Research Capsule**

What do we already know about this issue?

*Thoracic injury is the 2nd leading cause of death in blunt trauma. Computed tomography chest (CCT) is more sensitive than radiograph but doesn’t always spur change in management.*

What was the research question?

*Do older children undergo more changes in management than younger children screened for thoracic injury with CCT?*

What was the major finding of the study?

*Diagnoses on CCT prompted surgical management in 0.9% of patients 13 years and under, in contrast to 4.7% of patients 14–17 years.*

How does this improve population health?

*Assessing the impact of diagnoses made by CCT on clinical and surgical management by pediatric age groups will help clinicians tailor their use of CCT.*

The registry identifies trauma patients presenting to the ED and assigns them a registry number. Registrars abstract patient medical records for 65 data points containing such elements as vitals, dates and times, prehospital data, procedures, comorbidities, outcomes, and financial data. The validation process is rigorous and includes audits of up to 10% of each trauma registrar by the trauma registry manager, physician investigator, nurses, and trauma program managers. In addition, data abstraction forms, software-related validation, feedback to the trauma registrars, and educational courses, including workshops on Abbreviated Injury Scale-ISS coding and scoring, ensure reliable data collection.

We did not analyze patients who only had CCT performed without a CXR or those who had CXR occurring after the CCT, as the latter may have influenced the reading on the CXR. While some children had a pan CT scan (a wide field-of-view CT imaging protocol from the head to the pubic symphysis) during this time period, some received the CCT only after a screening CXR. This was clinician dependent. We excluded patients with penetrating injury or injuries occurring greater than 24 hours prior to admission. As age of the injury could alter the appearance on the imaging study introducing bias, we focused on acute injuries. While it is difficult to isolate patients who specifically experienced blunt chest trauma, we obtained the total number of blunt trauma patients. We focused our analysis on patients definitively evaluated for chest trauma with CXR and CCT from all blunt trauma patients. We further subdivided the patients into five age groups: 0–2 years; 3–6 years; 7–10 years; 11–13 years; and 14–17 years to discern differences in diagnoses made on imaging and management changes based on differences in diagnoses by modality.

### Measurements and Outcomes

Four physician chart extractors queried the electronic health record (EHR) to determine specific diagnoses made on CXR and CCT and confirm the timing of any resulting procedures. Another physician reviewed 20% of these, and any discrepancy was settled by all parties. Images were read by radiologists from Memorial Herman Hospital. Change in management is the occurrence of a procedure resulting from a new diagnosis by CCT, not observed by the CXR. Outcome variables were diagnoses made by CCT as well as ensuing changes in clinical management attributable to the diagnoses reported by the CCT. The intrathoracic diagnoses categories were as follows: 1) contusion/atelectasis; 2) pneumothorax; 3) hemothorax/effusion; 4) rib fractures; 5) other fracture; 6) vascular injury; 7) mediastinal abnormality; 8) diaphragm rupture; 9) foreign body; and 10) incidental findings. The presence of each was determined on both CXR and CCT.

We also determined the types of clinical management changes, including surgical and medical changes, attributable to the CCT. These included the following: 1) chest tube; 2) surgical repair of fractures; 3) utilization of a brace, sling or non-weight-bearing (NWB), corset; 4) additional imaging such as magnetic resonance imaging (MRI) of specific body parts, esophograms, aortic angiograms, and skeletal radiographs; (5) surgical vascular repair; 6) medical vascular repair (therapeutic anticoagulation); 7) removal of a foreign body; 8) follow-up/referral to a specialist for incidental finding on CCT (ie, cardiology, oncology, pediatric surgery); and 9) surgical repair of the diaphragm. When any of these situations were present along with a discrepancy between diagnoses found on CXR and CCT, we considered it a change in management.

The independent variables were patients’ demographic and clinical characteristics. We collected information on age, gender, race/ethnicity, ISS, systolic blood pressure (SBP), heart rate, and GCS measured at arrival to the ED, as well as information on the mechanism of injury, and disposition from the ED. We further classified SPB and heart rate as hypotension and tachycardia, respectively for age, using Pediatric Advanced Life Support guidelines. We classified the mechanism of injury as MVC, including motorcycle and all-terrain vehicles, pedestrian or cyclist struck by a vehicle (auto-ped), falling, sports, machinery (exposure to inanimate mechanical forces as described in ICD-10 W20–W49 such as struck by, thrown, projected or falling object, contact with nonpowered hand tool, and explosion and rupture of other specified pressurized devices), and assault as well as non-accidental trauma. Emergency department disposition included admission to the hospital, admission to the intensive care unit, disposition to the operating room for surgery, discharge to home, or death in the ED.

We conformed to the methods of proper medical review studies.^18^ All data abstractors used a standardized instrument to collect injury data and clinical and surgical procedures from the EHR. Abstractors were trained in determining the time course of the intervention in relation to the CXR and CCT. Inclusion and exclusion criteria were strictly defined, and categorization of injury and types of procedures were determined in advance. We monitored research abstractors for accuracy by double checking data entry. Data abstractors were aware of the original study hypothesis, that CCT would discover more injuries than CXR but would only change clinical and surgical management in a minority of patients, but they were not aware of the secondary analysis hypothesis that older children would undergo most of the clinical and surgical management changes in comparison to younger children. The trauma database and its validation process are described above, and the IRB approved the study as stated.

### Data Analysis

We determined the characteristics of patients in each age group including demographics, injury mechanism, and hospital disposition. We determined the rates and types of clinical and surgical management changes across different age groups. To quantify the variations in CT diagnosis, change in clinical management and change in surgical management across age groups, we used logistic regression. We fit separate models for the dependent variables: CT diagnosis; change in clinical management; and change in surgical management. The independent variable was patient age, which we classified into five pediatric age groups: 0–2 years; 3–6 years; 7–10 years; 11–13 years; and 14–17 years. For each age category, we determined the odds ratio for CT diagnosis, change in clinical management, and change in surgical management. We repeated the analysis adjusting the model for injury mechanism (fall, MVC, and other), ISS, and GCS.

## RESULTS

There were 1,235 patients with blunt trauma evaluated by both CCT and CXR meeting inclusion criteria from 8,283 similarly aged patients presenting with blunt trauma over the study time period. Table 1 shows the total number of blunt trauma patients seen at our facility per age group and the percentages of those scanned with both CCT and CXR for intrathoracic injury.

Table 2 shows the patient demographics of those screened with both imaging modalities. Gender, race/ethnicity, disposition from ED, and ISS were relatively stable across age groups. Mechanism of injury shifted across age groups with falls/sporting injuries occurring most commonly in the youngest two age groups and assaults in the oldest age group (*P* = <0.01.) The MVC/motorcycle injuries were most common in the two oldest age groups. A GCS ≤ 12 and hypotension for age were present in the highest percentages in the youngest age groups, but only GCS ≤ 12 was statistically significant (P = < 0.01). Tachycardia was most common in the two oldest age groups (P = < 0.01).

When the five age groups were compared, the unadjusted and adjusted ORs of undergoing any clinical management changes were lower across younger age groups but only statistically significant for the 3–6 year group (unadjusted OR 0.4; confidence interval [CI]: 0.2–0.9); adjusted OR 0.5; CI: 0.2–1.0.) See Table 3a. Similarly, the odds of having a surgical management change were lower in the younger age groups but only statistically significant for the 3–6 year group (unadjusted OR 0.1; 95% CI: 0.0–0.7; adjusted OR 0.1; 95% CI: 0.0–0.9.) See Table 3a.

The use of CCT in making additional diagnoses when compared to CXR did not appear to change across age groups. Confidence intervals were either wide or crossed one (Table 3b). Of note, we did not include a statistical analysis for vascular injury, foreign body, and diaphragm rupture due to the small numbers found on CCT. There were 13 vascular injuries found on CCT in our total cohort, three in the 11–13 year age group and 10 in the 14–17 year age group. There was one diaphragmatic hernia diagnosis made on CCT in the 14–17 year age group. For foreign bodies, CCT diagnosed one in the 3–6 year age group, three in the 7–10 year age group, and one in the 11–14 year age group. The trend test across consecutive ages demonstrated an unadjusted and adjusted OR of undergoing any clinical management change of 1.1 (95% CI: 1.0–1.1; *P*-value < 0.01) and 1.1 (95% CI: 1.0–1.1; *P-*value 0.01), respectively. There was 10% increased odds of undergoing a change in management with each subsequent year of life. Similarly, the trend test across consecutive ages demonstrated an unadjusted and adjusted OR of undergoing a surgical management change of 1.3 (95% CI: 1.1–1.4; *P*-value <0.01) and 1.2 (95% CI: 1.1–1.4; *P-*value < 0.01), respectively. There was 20% increased odds of undergoing a surgical change in management with each subsequent year of life. The trend test did not show a difference in the odds of CCT finding specific lesions not captured on CXR across consecutive age groups (Table 4).

The frequency of surgical and nonsurgical changes in management increased with age (Table 5). The leading surgical changes were chest tubes and surgical repairs for fractures, followed by surgical repairs for vascular injuries. The percentages of surgical management changes within each age cohort increased with age across the five groups (0%, 0.5%, 1.1%, 1.5%, and 4.9%). The majority of nonsurgical changes in management were use of a brace followed by sling or non-weight bearing; corset; and more imaging. The oldest age group contained over half of all non-surgical management changes.

## DISCUSSION

The clinical impact of CCT according to pediatric age group has not been determined in previous studies. In our study, older age groups as opposed to younger age groups underwent more evaluations with CCT than with CXR alone. The detection of important injuries and ensuing clinical and surgical changes in management were also more likely to occur in older age groups. Most of those surgical management changes occurred in the oldest of the five subgroups of age. Over half of any clinical management changes occurred in the oldest age group. Trauma centers use different age cutoffs ranging from 2–21 years of age for designation of pediatric trauma vs adult trauma; the optimal inflection point is not known. The age cutoff is important as it often determines the types of screening modalities a patient will receive, guided by either trauma surgeons or pediatric surgeons. Knowing the impact of CCT in age groups and individual ages is critical to help better inform that distinction while recognizing that the decision is often locally resource driven. While many freestanding children’s hospitals screen children with CXR, the vast majority of children evaluated at general EDs may be at risk of getting the pan CT scan.

Prior studies show lower trends in CT utilization in patients <14 years than those 15–54 years of age.^19^ Roudsari and colleagues reported an increasing trend in pediatric CCT between 1996–2005, but utilization leveled between 2005–2010. Korley’s analysis of nationwide data using the National Hospital Ambulatory Medical Care Survey, on the contrary, demonstrated an increased trend in the overall use of CT and MRI in children aged 3–18 years from 1998–2007.^20^ Yet there was not an equal rise in the prevalence of diagnoses of life-threatening disorders or in the disposition of patients. Details regarding the nature of these injuries and the presence of clinical clues prior to imaging were missing in this analysis. Another study examining CT use for patients with abdominal pain also demonstrated an increase in utility without an increase in the rate of diagnosis of significant intra-abdominal conditions.^21^ A query of the National Trauma Data Bank from 2014 to 2016 of children under 14 years discovered CCT utilization occurred in 3%, 13%, and 22% of children when they had no injuries, minimal injury, and moderate injury to the chest, respectively.^22^ Level 1 stand-alone pediatric centers displayed significantly lower CT utilization rates than others. Our trauma center is a mixed trauma center, and the majority of patients seen annually are adults. Until June 2016, CCT was still employed in chest trauma screening. Since then, children 15 years and under receive a CXR unless there is a concern for a widened mediastinum. The 16 years and older group may undergo either modality.

Our prior analysis demonstrated that while CCT may diagnose more lesions, only 8.7% of the study group experienced any change in clinical management and 2.6% experienced a change in surgical management due to CCT.^23^ When the total blunt trauma population makes up the denominator, those percentages drop to 1.3% and 0.37%, respectively. Furthermore, a closer analysis of patient clinical characteristics for the “other fractures,” and the vascular injuries found on CCT resulting in surgical change in management, had physical exam findings, low GCS or unstable vitals, which would have prompted further imaging or investigations.^10^ Our data is in stark contrast to Langdorf et al.’s findings in adults when analyzing occult injuries found in CCT and not on CXR.^24^ They found up to 25% occult injuries on CCT and of those, 14% and 24% resulting in major and minor interventions, respectively.

Chest computed tomography may expose a pediatric patient to 1.5 millisievert (mSV) if the child is less than age five years and over 8 mSV when the child is over 10 years of age.^25^ The known long-term sequelae of exposing the developing child to ionizing radiation has led to increased efforts to appropriately diagnose trauma-related injuries and minimize radiation exposure in children when possible.^26^ The use of CCT should also include consideration of charges and costs to patient, insurers, and society. At our teaching facility, the technical and professional charge for a CCT with contrast is $4,262 and $385, respectively. Currently, medical imaging is one of the costliest diagnostic techniques and the most used. Imaging machines are very expensive; a new, higher slice CT machine costs as much as $2.5 million not including the recurring maintenance and equipment fees. Aside from these charges, obtaining these studies adds to the LOS to obtain images and await their readings. The overwhelming majority of the studies we performed in children in our cohort made no difference to their management, but this was especially true for the younger children. Moreover, CCT may actually prolong LOS, contributing to an inefficient healthcare system. In a recent analysis, the liberal utilization of CT did not lead to a quicker discharge home, and more than four CTs were independently associated with longer LOS independent of ISS.^5^ In addition, when scanning the cervical spine or the chest, there was at least one false positive result for every two clinically significant findings obtained, calling into question the practice of liberal imaging of these regions.^5^

Ultimately, readers will differ in opinion as to what delineates significant thoracic trauma as there are no widely accepted delineations or gauges of magnitude. Inter-specialty differences of opinion about the clinical implication of specific injuries exist.^27^ While minor missed injuries may heal on their own, ones that are more critical may become evident with repeated exams and observation, strict return precautions, and appropriate follow-up. In the pursuit of a zero missed-injury rate, we must weigh the risk beyond cost, time, and resources with the threat of iatrogenic cancer and contrast-induced nephropathy. Clear and respectful communication with patients and families and shared decision-making with proper documentation are key to balancing the risks and benefits of trauma imaging.

The American College of Surgeons has historically used age 15 as the cutoff between adult and pediatric trauma designation based upon physiological and anatomic estimates. The age cutoff may vary depending on local resources within a hospital system. This is important as imaging protocols are often developed separately for pediatric and adult patients. Information about the outcomes associated with CCT is pivotal in judicious imaging practices and limiting radiation exposure to more vulnerable populations.

## LIMITATIONS

First, because our study was retrospective some information from the EHR and trauma registry may have been more susceptible to misclassification and bias compared to prospective studies. Our study represents pediatric patients treated at our mixed trauma facility in Houston, Texas, which sees both adults and children. Our results may not be generalizable to other institutions locally or nationally with differing proportions of age groups in their centers. However, we do have the influence of both general trauma surgeons as well as pediatric surgeons, as experienced at different centers. Studies have shown higher rates of CT usage at adult and mixed trauma centers vs pediatric trauma centers.^28^–^30^

Our categories of changes in management secondary to CCT may not encompass factors that other clinicians may deem important. Some would add to our list and include admission to hospital for observation, as a significant change in management. Furthermore, we defined a change in management resulting from a procedure performed after the CCT in the setting of a discordance of diagnoses between the two imaging modalities. This has the inherent risk of overselling the impact of CCT. It is also possible there was a bias toward change in management given the overdependence on CCT. None of the 128 patients in the 0–2 years age group who received a CCT had a surgical change in management. However, there were far fewer patients in this category than in the older age groups, limiting the power of the observation. These limitations naturally occur with retrospective studies.

## CONCLUSION

We demonstrate that young children seldom undergo management changes when additional diagnoses occur on chest computed tomography in pediatric trauma. Practitioners should be thoughtful about automatically obtaining a CCT on the young, multi-trauma patient.

## Figures and Tables

**Table 1:** Proportion of pediatric blunt trauma patients undergoing screening for intrathoracic injury.

	Ages 0–2	Ages 3–6	Ages 7–10	Ages 11–13	Ages 14–17	Total
All blunt trauma patients, N	1,744	1,826	1,228	883	1,364	8,283
All blunt trauma patients undergoing CXR and CCT, N (%)	128 (7.3)	212 (11.6)	175 (14.3)	188 (21.0)	532 (39.0)	1,235 (15.0)

*CXR*, chest radiograph; *CCT*, chest computed tomography.

**Table 2:** Patient characteristics by age group.

	Age group					
		
	0–2 years N = 128 median (IQR)	3–6 years N = 212 median (IQR)	7–10 years N = 175 median (IQR)	11–13 years N = 188 median (IQR)	14–17 years N = 532 median (IQR)	*P*-value a
		
	1.7 (1.1 – 2.0)	4.0 (4.0 – 6.0)	8.0 (7.0 – 9.0)	12.0 (12.0 – 13.0)	16.0 (15.0 – 17.0)	
Gender, N (%)						0.44
Female	58 (45.3)	91 (42.9)	71 (40.6)	72 (38.3)	200 (37.6)	
Male	70 (54.7)	121 (57.1)	104 (59.4)	116 (61.7)	332 (62.4)	
Race, N (%)						0.10
Asian	0 (0.0)	6 (2.8)	6 (3.4)	4 (2.1)	8 (1.5)	
Black	34 (26.6)	31 (14.6)	35 (20.0)	33 (17.6)	91 (17.1)	
Hispanic	57 (44.5)	80 (37.7)	61 (34.9)	67 (35.6)	193 (36.3)	
Other	2 (1.6)	5 (2.4)	3 (1.7)	2 (1.1)	9 (1.7)	
White	35 (27.3)	90 (42.5)	70 (40.0)	82 (43.6)	231 (43.4)	
Mechanism, N (%)						< 0.01
Assault/non-accidental	0 (0.0)	0 (0.0)	0 (0.0)	0 (0.0)	12 (2.3)	
Fall, sporting, machine	43 (33.6)	30 (14.2)	15 (8.6)	10 (5.3)	47 (8.8)	
Motor vehicle, motorcycle	54 (42.2)	140 (66.0)	115 (65.7)	132 (70.2)	387 (72.7)	
Pedestrian/bicycle	31 (24.2)	42 (19.8)	44 (25.1)	46 (24.5)	86 (16.2)	
Disposition, N (%)						0.11
Admitted to hospital	49 (38.3)	92 (43.4)	80 (45.7)	80 (42.6)	238 (44.7)	
Admitted to ICU	65 (50.8)	93 (43.9)	65 (37.1)	74 (39.4)	194 (36.5)	
Death	0 (0.0)	0 (0.0)	0 (0.0)	2 (1.1)	1 (0.2)	
Home	0 (0.0)	0 (0.0)	0 (0.0)	0 (0.0)	3 (0.6)	
Surgery	14 (10.9)	27 (12.7)	30 (17.1)	32 (17.0)	96 (18.0)	
Injury Severity Score, N (%)						0.15
< 15	53 (41.4)	113 (53.3)	92 (52.6)	84 (44.7)	259 (48.7)	
≥ 15	75 (58.6)	99 (46.7)	83 (47.4)	104 (55.3)	273 (51.3)	
Glasgow Coma Score, N (%)						0.01
> 12	72 (56.3)	126 (59.4)	120 (68.6)	125 (66.5)	377 (70.9)	
≤ 12	54 (42.2)	82 (38.7)	55 (31.4)	61 (32.4)	151 (28.4)	
Hypotension, N (%)						0.29
Yes	8 (6.3)	8 (3.8)	3 (1.7)	6 (3.2)	17 (3.2)	
No	114 (89.1)	204 (96.2)	169 (96.6)	182 (96.8)	513 (96.4)	
Tachycardia, N (%)						< 0.01
Yes	12 (9.4)	33 (15.6)	15 (8.6)	93 (49.5)	242 (45.5)	
No	116 (90.6)	179 (84.4)	160 (91.4)	95 (50.5)	290 (54.5)	

a *P*-value for hypotension was derived from Fisher’s exact test. All other p-values were derived from Pearson’s chi-square test.

*IQR*, interquartile range; *ICU*, intensive care unit.

**Table 3a:** Associations between age and changes in clinical and surgical management.

	N/Subgroup (%)	Unadjusted odds ratio (95% CI)	Adjusted odds ratio (95% CI) a
Management change			
0–2 years	5/128 (3.9)	0.3 (0.1 – 0.8)	0.4 (0.2 – 1.1)
3–6 years	11/212 (5.2)	0.4 (0.2 – 0.9)	0.5 (0.2 – 1.0)
7–10 years	16/175 (9.1)	0.8 (0.5 – 1.5)	1.0 (0.5 – 1.8)
11–13 years	17/188 (9.0)	0.8 (0.5 – 1.4)	0.8 (0.4 – 1.4)
14–17 years	58/532 (10.9)	Reference^b^	Reference^b^
Surgical management change			
0–2 years	0/128 (0.0)	–	–
3–6 years	1/212 (0.5)	0.1 (0.0 – 0.7)	0.1 (0.0 – 0.9)
7–10 years	2/175 (1.1)	0.2 (0.1 – 1.0)	0.3 (0.1 – 1.3)
11–13 years	3/188 (1.6)	0.3 (0.1 – 1.1)	0.3 (0.1 – 1.1)
14–17 years	25/532 (4.7)	Reference^b^	Reference^b^

a The adjusted odds ratios were adjusted for injury mechanism (fall, motor vehicle collision, and other), injury severity score, Glasgow Coma Scale, hypotension, and tachycardia.

b Refers to reference used to calculate the odds ratios.

*CI*, confidence interval.

**Table 3b:** Association between age and positive chest computed tomography findings.

	N (Positive diagnosis^a^)/(subgroup) (%)	N (CCT only)/(positive diagnosis^a^) (%)	Unadjusted odds ratio (95% CI)	Adjusted odds ratio^b^ (95% CI) c
Pneumothorax				
0–2 years	21/128 (16.4)	15/21 (71.4)	1.6 (0.6 – 4.4)	1.8 (0.6 – 5.7)
3–6 years	41/212 (19.3)	24/41 (58.5)	0.9 (0.5 – 1.9)	0.8 (0.4 – 1.6)
7–10 years	36/175 (20.6)	27/36 (75.0)	1.9 (0.8 – 4.4)	1.6 (0.6 – 3.8)
11–13 years	51/188 (27.1)	34/51 (66.7)	1.3 (0.7 – 2.5)	1.3 (0.7 – 2.7)
14–17 years	140/532 (26.3)	85/140 (60.7)	Reference^d^	Reference^d^
Hemothorax/effusion				
0–2 years	2/128 (1.6)	0/2 (0.0)	–	–
3–6 years	1/212 (0.5)	1/1 (100.0)	–	–
7–10 years	10/175 (5.7)	7/10 (70.0)	1.4 (0.3 – 6.7)	1.3 (0.2 – 7.9)
11–13 years	11/188 (5.9)	9/11 (81.8)	2.8 (0.5 – 15.1)	3.8 (0.6 – 26.5)
14–17 years	29/532 (5.5)	18/29 (62.1)	Reference^d^	Reference^d^
Other fractures				
0–2 years	13/128 (10.2)	6/13 (46.2)	0.6 (0.2 – 1.9)	0.8 (0.2 – 2.8)
3–6 years	36/212 (17.0)	19/36 (52.8)	0.8 (0.4 – 1.7)	0.8 (0.4 – 1.8)
7–10 years	39/175 (22.3)	18/39 (46.2)	0.6 (0.3 – 1.2)	0.7 (0.3 – 1.4)
11–13 years	49/188 (26.1)	25/49 (51.0)	0.7 (0.4 – 1.4)	0.8 (0.4 – 1.6)
14–17 years	126/532 (23.7)	74/126 (58.7)	Reference^d^	Reference^d^
Mediastinal abnormality				
0–2 years	6/128 (4.7)	3/6 (50.0)	1.9 (0.3 – 10.3)	3.2 (0.4 – 27.9)
3–6 years	11/212 (5.2)	6/11 (54.5)	2.3 (0.6 – 8.4)	1.9 (0.5 – 7.9)
7–10 years	9/175 (5.1)	3/9 (33.3)	0.9 (0.2 – 4.2)	0.9 (0.2 – 4.7)
11–13 years	19/188 (10.1)	7/19 (36.8)	1.1 (0.4 – 3.3)	0.9 (0.3 – 3.0)
14–17 years	55/532 (10.3)	19/55 (34.5)	Reference^d^	Reference^d^
Incidental finding				
0–2 years	9/128 (7.0)	8/9 (88.9)	2.3 (0.3 – 20.6)	2.1 (0.2 – 21.4)
3–6 years	6/212 (2.8)	5/6 (83.3)	1.5 (0.2 – 13.8)	1.2 (0.1 – 13.4)
7–10 years	7/175 (4.0)	6/7 (85.7)	1.8 (0.2 – 16.0)	2.4 (0.2 – 26.5)
11–13 years	19/188 (10.1)	14/19 (73.7)	0.8 (0.2 – 2.7)	0.7 (0.2 – 2.5)
14–17 years	53/532 (10.0)	41/53 (77.4)	Reference^d^	Reference^d^

a Positive diagnosis consisted of total number of patients who had positive diagnosis in either CCT or chest radiograph (CXR).

b The odds in the logistic regression model had CCT only as numerator and all other positive imaging diagnoses (CXR only, and both CCT and CXR) as denominator.

c The adjusted odds ratios were adjusted for injury mechanism (fall, motor vehicle collision, and other), Injury Severity Score, Glasgow Coma Scale, hypotension, and tachycardia.

d Refers to reference used to calculate the odds ratios.

*CCT*, chest computed tomography; *CI*, confidence interval.

**Table 4:** Association between age and changes in management, surgical management and positive chest computed tomography findings.

	Unadjusted		Adjusted^a^	
		
	Odds ratio^b^ (95% CI)	*P*-value	Odds ratio^b^ (95% CI)	*P*-value
Management change	1.1 (1.0 – 1.1)	< 0.01	1.1 (1.0 – 1.1)	0.01
Surgical management change	1.3 (1.1 – 1.4)	< 0.01	1.2 (1.1 – 1.4)	< 0.01
Positive chest CT findings				
Pneumothorax	1.0 (0.9 – 1.0)	0.54	1.0 (0.9 – 1.0)	0.87
Hemothorax effusion	1.0 (0.9 – 1.2)	0.80	0.9 (0.8 – 1.1)	0.55
Other fractures	1.0 (1.0 – 1.1)	0.13	1.0 (1.0 – 1.1)	0.30
Mediastinal abnormality	1.0 (0.9 – 1.0)	0.31	1.0 (0.9 – 1.1)	0.45
Foreign body	0.9 (0.8 – 1.1)	0.37	0.9 (0.7 – 1.1)	0.35
Incidental finding	0.9 (0.8 – 1.0)	0.22	0.9 (0.8 – 1.1)	0.32

a The adjusted odds ratios were adjusted for injury mechanism (fall, motor vehicle collision, and other), Injury Severity Score, Glasgow Coma Scale, hypotension, and tachycardia.

b Odds ratio for each additional year of age.

*CT*, computed tomography; *CI*, confidence interval.

**Table 5:** Frequencies of changes in management by age group.

Management code, N (%)	Age group				

	0–2 years	3–6 years	7–10 years	11–13 years	14–17 years
Surgical management					
Chest tube	0 (0.0)	0 (0.0)	2 (1.1)	1 (0.5)	11 (2.1)
Surgery for fracture	0 (0.0)	0 (0.0)	0 (0.0)	0 (0.0)	9 (1.7)
Surgical vascular repair	0 (0.0)	0 (0.0)	0 (0.0)	1 (0.5)	5 (0.9)
Foreign body removal	0 (0.0)	1 (0.5)	0 (0.0)	1 (0.5)	0 (0.0)
Diaphragm repair	0 (0.0)	0 (0.0)	0 (0.0)	0 (0.0)	1 (0.2)
Non-Surgical management					
Brace	2 (1.6)	5 (2.4)	5 (2.9)	7 (3.7)	14 (2.6)
Sling, or NWB; corset	1 (0.8)	2 (0.9)	6 (3.4)	5 (2.7)	14 (2.6)
More imaging	1 (0.8)	3 (1.4)	5 (2.9)	2 (1.1)	11 (2.1)
Medical vascular repair (therapeutic anticoagulation)	0 (0.0)	0 (0.0)	0 (0.0)	1 (0.5)	2 (0.4)
Specialist follow-up	1 (0.8)	1 (0.5)	0 (0.0)	0 (0.0)	2 (0.4)

*NWB*, non weight-bearing.
